# The Effect of Financial Scarcity on Reinforcer Pathology: A Dyadic Developmental Examination

**DOI:** 10.3390/children9091338

**Published:** 2022-09-01

**Authors:** Amanda K. Crandall, Leonard H. Epstein, Jennifer Fillo, Kevin Carfley, Eleanor Fumerelle, Jennifer L. Temple

**Affiliations:** 1 Department of Community Health and Health Behavior, School of Public Health and Health Professions, University at Buffalo, 3435 Main Street, Buffalo, NY 14214, USA; 2 Department of Pediatrics, Jacobs School of Medicine, University at Buffalo, 3435 Main Street, Buffalo, NY 14214, USA; 3 Department of Health Promotion, Education, and Behavior, Arnold School of Public Health, The University of South Carolina, 915 Greene St Discovery I, Suite 551, Columbia, SC 29208, USA; 4 Department of Exercise and Nutrition Sciences, School of Public Health and Health Professions, University at Buffalo, 3435 Main Street, Buffalo, NY 14214, USA

**Keywords:** food insecurity, scarcity, reinforcer pathology, food reinforcement, delay discounting

## Abstract

This study investigated the effects of experimentally manipulated scarcity on the reinforcing value of food (RRV_food_) and delay discounting (DD), which, together, create reinforcer pathology (RP) among parents and offspring. A stratified sample of 106 families (53 parent/child aged 7–10 dyads & 53 parent/adolescent aged 15–17 dyads) from high- and low-income households visited our laboratory for three appointments. Each appointment included an experimental manipulation of financial gains and losses and DD and RRV tasks. The results showed that, regardless of food insecurity or condition, children had greater RP (β = 1.63, *p* < 0.001) than adolescents and parents. DD was largely unaffected by acute scarcity in any group, but families with food insecurity had greater DD (β = −0.09, *p* = 0.002) than food-secure families. Food-insecure parents with children responded to financial losses with an increase in their RRV_food_ (β = −0.03, *p* = 0.011), while food-secure parents and food-insecure parents of adolescents did not significantly change their responding based on conditions. This study replicates findings that financial losses increase the RRV_food_ among adults with food insecurity and extends this literature by suggesting that this is strongest for parents of children.

## 1. Introduction

Food insecurity is the state of being without reliable access to a sufficient quantity of affordable, nutritious foods [1]. This experience ranges from anxiety over one’s financial ability to obtain food for all household members to, at the most severe level, a disruption in eating patterns and reduced food intake [2]. Food insecurity is associated with a greater risk of chronic disease, including obesity, and reduced life expectancy [3,4,5,6]. Due to lower costs [7] and limited access to fresh fruits and vegetables [8,9], people with food insecurity tend to have more energy-dense foods in the home [10,11] and children growing up in food-insecure homes have a greater risk of excess energy intake and higher rates of obesity [5,12]. Experiences of stress and poverty in childhood are also associated with adult obesity [13,14]. Recent evidence suggests that having limited financial resources can have direct effects on eating behavior by increasing food motivation and decreasing impulse control, a construct known as reinforcer pathology (RP) [15], but less is known about how poverty in childhood may impact eating behavior over the life course.

The relative reinforcing value of energy-dense foods (RRV_food_) is the amount of work one is willing to put into obtaining a portion of such food [16]. A greater RRV_food_ predicts greater energy intake, both in the laboratory [17] and in daily life [18], and it is an independent risk factor for obesity among adults and children [19,20,21]. The RRV_food_ mediates the relationship between socioeconomic status (SES) and body mass index (BMI) in adults [22]. Food insecurity is associated with the RRV_food_ in pregnant women [23], and food-insecure adults have a greater RRV_food_ after financial losses compared with food-secure adults [24]. Likewise, household income is associated with a greater RRV_food_ among adolescents [25]. This evidence suggests that household resources, and food insecurity specifically, may impact the reinforcing value of food among adults and adolescents, but more work is needed to assess the causal nature of these associations and their developmental impact.

The other facet of RP is the tendency to prefer smaller immediate rewards over larger but delayed rewards or delay discounting (DD) [26,27]. People who value immediate reinforcers, such as food, as evidenced by steep discounting rates, are more likely to be obese, consume more calories in ad libitum eating tasks, and lose less weight in weight control programs [15]. Navigating the many structural barriers that exist for people living in poverty causes greater stress [28,29], cognitive loads [30,31], and depression [32], all of which increase DD [33,34,35,36]. Likewise, the narrowing of the temporal window associated with food insecurity tends to cause adults to focus on immediate reinforcers, including food [37]. Indeed, DD is cross-sectionally related to financial resources in both adults [38,39] and children [40], and scarce financial resources impact DD developmentally [41]. Priming participants with a hypothetical financial crisis increases both food demand and DD in adults with obesity [34]. Evidence from toddlers suggests that prioritizing immediate needs is adaptive in the context of poverty [42], but little experimental and longitudinal work has been done among children and adolescents to understand the extent to which the environment of household food insecurity may affect DD. Research is needed to examine the extent to which changes in financial resources may affect RP in youth.

Familial relationships can impact eating behavior through a shared food environment in the home [10] as well as the decisions that parents make when feeding their children [13,43]. Depression and family stress, which are associated with food insecurity, both impact parental feeding practices [44,45]. Parents with food insecurity also report a range of strategies to protect their children from the harmful effects of food insecurity, particularly hunger, including reducing their own portions of food so the child may eat more [43] and choosing well-liked, energy-dense foods to ensure the child will not feel hungry [46]. However, children as young as four years old understand and react to scarce resources in a similar manner to adults [44,45], but little is known about how food insecurity during childhood may impact the development of eating behavior— in particular, RP. Adolescents independently experience food insecurity [47], but few studies have examined how it may affect their RP. While the RRV_food_ is associated between parents and children, DD is not [48]. However, familial SES is associated with elevated DD for both children and their parents [39,40,42,49,50]. More research is needed to examine the effects of food insecurity and household resources on RP in childhood and adolescence, with particular attention to the family unit, as food insecurity occurs at the household level when offspring are involved [2,51].

The current study sought to examine the associations among acute scarcity (manipulated in the laboratory), food insecurity, and RP across the socioeconomic spectrum and within families. The use of family dyads allows for the control of unmeasured household characteristics and the assessment of household level versus individual level differences. We investigated families with either an elementary-aged child (7–10 years) or an older adolescent (15–17 years). These ages were chosen in order to examine differences in families in which the parent has a larger degree of control over the offspring food environment and those in which the offspring is more independent as they near adulthood [52] as well as more likely to experience food insecurity directly [47]. Based on the previous research noted above [48], we hypothesized that the RRV_food_, but not DD, would be associated between parents and offspring, regardless of age. As has been shown in previous research [53], we hypothesized that age would be associated with DD such that children would be the most present-focused, followed by adolescents, and that parents would be the most future-oriented. Based on these anticipated associations, we hypothesized that, within families, children would have significantly greater RP than their parents, while adolescents would not significantly differ. As shown previously among adults [25], we hypothesized that adults with food insecurity would be more sensitive to acute financial losses and respond with greater RP. Because adolescents are more likely to experience food insecurity directly, we hypothesized that the adolescents from food-insecure households would also be sensitive to acute financial losses and respond with greater RP, while children, who are more likely to be shielded from food insecurity by their parents, would show no difference in RP based on financial losses, regardless of food security status.

## 2. Methods

### 2.1. Study Participants

#### 2.1.1. Recruitment and Inclusion/Exclusion Criteria

The methods for this study have been described elsewhere [54,55]. All recruitments, study materials, and procedures were approved by the University at Buffalo institutional review board. Families with one adolescent aged 15–17 years or one child aged 7–10 years were recruited from the Western New York area using flyers placed around the community and sent directly to local elementary and high schools. The main purpose of the study was concealed from all advertisements. Particular effort was made to recruit from low-income zip codes to increase the number of food-insecure families who completed the screening survey. Parents interested in the study completed a screening survey, and eligible parents were contacted by the study team and scheduled for three separate visits to the laboratory at the University at Buffalo South Campus, with at least one full day between each appointment. The adolescent and child families were two separate groups of families, with no siblings included in the study. The inclusion criteria were families with an adolescent or child falling within the age requirement noted above and both the parent and the offspring rating the study foods as at least “slightly liked” and rating the granola bars available as at least “neither liked nor disliked” on a 5-point Likert type scale. In order to participate in the study, both the parent and the offspring needed to be willing to fast for 2 h prior to the appointment time, attend all three appointments, consume a granola bar, and participate in computerized tasks and surveys. Additionally, both the parent and the offspring needed to speak English, and parents needed to be able to read English to consent to the study. In the case of the children (7–10 years), study materials were read to them by the research assistants, as needed. The exclusion criteria were pregnancy, health conditions for which the participant reported effects on appetite and/or eating, and the use of stimulant medication or any medication because of which the parent reported changes to appetite. The data for this study were collected from November 1st, 2018, through February 28th, 2020. The study was completed before the Western New York area recorded their first COVID-19 case, so no adaptations to the study protocol were needed due to the COVID-19 pandemic.

#### 2.1.2. Sample Size Determination and Stratifying the Sample

This study was a within- and between-subjects nested design in which all participants, who were recruited in family pairs, experienced the same 3-level within-subject manipulation, and the responses were compared between subjects based on food security status. To protect participant comfort during screening, food insecurity was not assessed until participants enrolled in the study. Instead, participants were recruited based on the receipt of food assistance. The sample size for this study was based on our previous work in adults [24]. Given an effect size (f) of 0.31 for RRV_food_ between adults with and without food insecurity and across the within-subjects manipulation of financial gains and losses in that study and a power of 0.80, 16 individuals would be needed per group to detect a significant result at an alpha of 0.05. We planned to recruit 104 families, which consisted of 26 parent-child dyads and 26 parent-adolescent dyads within each assistance group. The sample was balanced by the biological sex of the offspring, and the groups were targeted such that the group receiving assistance had at least 30% of the participants reporting white race and the group reporting no assistance had at least 30% reporting non-white race for the offspring. The participating parent/guardian did not need to be biologically related to the offspring but did need to be a primary caregiver to the offspring.

### 2.2. Assessments

#### 2.2.1. BMI Measurement

We calculated the adults’ body mass index (BMI) from the measured weight (kg) and height (m) using the standard equation: kg/m^2^. For children and adolescents, we calculated a z-score (zBMI) based on a sex- and age-specific standard population, which reflects growth patterns in the United States [56].

#### 2.2.2. Manipulated Iowa Gambling Task

The Iowa Gambling Task is a well-validated computer-based task that measures risk-taking behavior in a laboratory [57]. To complete the traditional task, which was presented on computers via Inquisit (Millisecond, Seattle, WA, USA), participants made choice between different decks of cards. Each deck had different monetary wins and losses associated with it, and there was variability in the risk accompanying each deck. For 100 consecutive trials, participants chose between the decks and observed the changes in their total winnings as the task progressed [57]. For our purposes, participants started the task with USD 5 in all three conditions. The Control condition was the unmanipulated Iowa Gambling Task [57], which ended with participants typically breaking even with USD 5 in winnings. In the Gain and Loss conditions, participants were presented with the same four decks, which were comprised of the same risk of winning and losing money. However, the amounts won and lost were altered to bias the game toward larger wins or larger losses, accordingly. We have used this manipulation in our previous work to create real financial gains and losses for the participants as well as prime thoughts of financial losses through the continuous loss of money across 100 trials [24]. We improved the manipulation from our previous study [24] to better differentiate between conditions by increasing both the gains and losses. The games were pilot-tested by non-participating students and research assistants in our laboratory, who were unaware of the purpose or parameters of the task. The typical gain condition winnings were + USD 20, and the loss condition winnings were – USD 20. Participants were told at the beginning of the study that their winnings would be added together at the end of the study, and they would be able to take home any accumulated winnings. Their base payment for participation was unaffected by the losses on the task.

#### 2.2.3. Relative Reinforcing Value Task

Both the parents and offspring were asked to fast for two hours prior to attending their appointment and were given a granola bar before completing the RRV_food_ task in order to normalize hunger across the sample and limit its influence on the measurement of RRV_food_ [58,59]. Participants chose their study food and seated activity during the first appointment via self-ratings of available foods and activities. A well-liked food and alternative activity were used in order to ensure an accurate measurement of RRV_food_, which is associated with, but independent from, food liking [19,60]. These high-energy density foods were used for this protocol because the RRV of high-energy density foods is a risk factor for obesity in adults [19] and children [61].

To measure RRV_food_, participants completed a standardized reinforcing value task [62]. Participants sat at a desk with two computers and could work (i.e., click the mouse) for food portions on one and for seated activity time on the other. The available foods were M&M’s, Reece’s Pieces, plain potato chips, flavored tortilla chips, and Skittles. We provided a variety of seated activities, including age-appropriate magazines, drawing and art supplies, puzzles and activity books, and noncomputerized electronic games (e.g., Simon or electronic poker). The RRV task was visually similar to a slot machine, as each computer screen showed a set of three different colored shapes, and participants had to click the screen to rotate the shapes. They earned one point each time all shapes matched, and once five points were earned, participants were given a portion of their study food or two minutes for their seated activity, depending on which computer they chose. Participants could only play on one computer screen at a time but could switch back and forth as they pleased. The task became more difficult as the participant continued to play, with each round requiring more mouse clicks to earn a reinforcer. We used a progressive ratio schedule of reinforcement of 4, 8, 16, 32, 64, etc. Thus, to earn the first portion of food or activity time, participants needed to click the mouse but 20 times (+/−5%), 40 times for the second portion, 80 times for the third portion, etc. Upon completion of the task, participants were given time to eat the food portions they earned and then use their seated activity time, separately, if they wished. The participants were allowed to take home any food that they earned but did not eat in the laboratory. For the individual score for RRV of each reinforcer, the response requirement for the highest schedule completed (i.e., breakpoint) was used. These methods have been used in the past and are considered valid to measure the RRV_food_ [24,62].

#### 2.2.4. Delay Discounting Task

DD was assessed on each visit using an adjusting amount DD task [63] presented via Inquisit (Millisecond, Seattle, WA, USA). This asked participants to make choices between an immediate value of money and a larger but delayed amount of money. The task then adjusted the immediate value until it was subjectively equivalent to the later, larger amount, which is the indifference point. Indifference points were obtained at six delays: one day, one week, three months, six months, one year, and five years. The delays were kept consistent between the three age groups, but the delayed amounts were USD 1000, USD 100, and USD 50 for parents, adolescents, and children, respectively. We chose this difference in the delayed amounts in order to have a value that would be meaningful at each developmental stage. Indifference points across delays were removed if nonsystematic responses of 20% or more of the preceding delayed amount were observed [64]. We used the area under the curve of indifference points across the six delays to calculate an individual score for each participant [63].

#### 2.2.5. Household Food Insecurity Questionnaire

Parents answered questions about their own food insecurity as well as the overall household food insecurity levels using the USDA food security scale (Jilcott, Wall-Bassett, Burke, & Moore, 2011). For example, questions included, “I/We worried whether our food would run out before I/we got money to buy more” and “I/We relied on only a few kinds of low-cost food to feed the child because there wasn’t enough money for food”. Response options included, “Often true in the last 12 months, sometimes true in the last 12 months, never true in the last 12 months, I don’t know, or prefer not to answer”. We calculated the household food security score by summing affirmative responses and then categorized them into their standard categories (0 = Food-secure, 1−2 = Marginal food security, 3−7 = Low food security, > 7 = Very low food security) [65,66].

#### 2.2.6. MacArthur SES Questionnaire

To assess socioeconomic status, participants completed the MacArthur SES questionnaire, which consists of parent education level, household size, and household income, including government assistance, child support/alimony, and disability. Answer choices for income levels ranged from “Less than $5000” to “Over $100,000”. We used the midpoint of each income range as the value for household income in order to include this variable in correlation and regression analyses. We calculated income per person by dividing the total income by household size and using this value to determine the poverty status according to the federal poverty line [67].

#### 2.2.7. Appetite Sensations and Activity Liking

During each of the three appointments, participants reported hunger, thirst, food liking, and food wanting for their chosen study food. Similar questions were asked for the liking and wanting of the seated activity for which they would be working. Ratings were completed using a 100 mm visual analogue scale. This method has been used in prior studies to examine current appetite sensations [24,25,68].

### 2.3. Study Procedures

All study procedures were approved by the University at Buffalo institutional review board. These procedures have been described elsewhere [54,55]. Across all appointments, parents and offspring completed the same study procedures, differing only in surveys designed for Adults vs. Children/Adolescents. The study consisted of three visits to our laboratory, the first of which included consent from the parent and assent from the offspring. Trained research assistants showed families a standard video that explained the study procedures, including what participants would be asked to do, and their rights to refuse participation and/or study procedures. Specifically, the families were informed that they could refuse any part of the study without penalty and could withdraw from the study at any time and still be paid for the appointments they attended. The nature of the research questions was concealed from participants, and they were not told that the appointments differed by condition. Two experimental rooms with an adjoining door were used to accommodate families and allow for privacy between parents and offspring while still ensuring that they were close enough to feel comfortable. Within experimental rooms, participants sat at a computer station, while experimenters sat behind a divider at a control computer.

Immediately after consent, height and weight measurements were taken for both the parent and the offspring. Weight measurements were taken without shoes and after the removal of heavy clothing—in kg, using a SECA scale. Height measurements were taken in triplicate using a SECA stadiometer. In the case of participants with hairstyles that could not be easily taken down, a ruler was used to measure the hair, and this was then subtracted from the initial height measurement. The parents and offspring were kept together for the height and weight measurements and then separated in the adjoined rooms for the remaining phases of the appointment. The offspring, particularly the children, were encouraged to step back into the room with their parents as often as they wanted.

After separating, participants chose a granola bar flavor, a high-energy density food (for the RRV task), and a sedentary activity that remained constant throughout all appointments. A same-day food recall interview was then implemented using the five-pass method to confirm 2 h of fasting prior to the appointment. Those who violated the fasting period were asked to wait for the necessary amount of time, or their appointments were rescheduled based on parent preference. Next, the participants’ hunger, thirst, and food and activity liking were assessed. Following this, participants were instructed to eat their granola bar and sit quietly while watching a 10 min video designed for meditation, which featured nature scenes and soothing music. This phase was designed to allow hunger to normalize before beginning the next phases of the appointments. After completing an additional assessment of hunger, thirst, and food/activity liking, the parents and offspring underwent the scarcity manipulation, which entailed a financial gain, loss, or neutral outcome (one condition per appointment). The order of the appointments was counterbalanced between participants, and the order was randomly assigned; therefore, the parents and offspring did not have the same manipulation condition on the same day.

Following the manipulation, participants completed the DD task. A second assessment of hunger, thirst, and food/activity liking was then administered prior to the RRV task. After the participant announced that they were finished playing for reinforcers, they were given the opportunity to eat their earned food and then, separately, use their activity time. A hunger, thirst, and food/activity liking assessment was administered again regardless of food or water consumption. Following this, participants completed survey assessments, which were spread across the three visits to lower participant burden. Those pertaining to economic position and food insecurity were administered in the final visit so as not to arouse suspicion in the participants.

After all the surveys were completed, the parents and offspring were brought back into the same room and were paid for that appointment. The total payment for the three appointments was USD 70 dollars for each participant plus any additional money they earned on the IGT (M = USD 8.55). After the final appointment, they were debriefed together. They were given a written explanation of the research questions, and the research assistants further explained the nature of the study and their rights to remove their data if they wished. Participants were given the opportunity to ask any remaining questions about the study and their participation.

### 2.4. Analytic Plan

We conducted all analyses using SPSS 26. Group differences between adolescent and child families as well as between those with and without food security were assessed with one-way analysis of variance in the case of continuous variables and chi-squared tests for categorical variables. For hypothesis testing, we used multilevel modeling using the MIXED procedure [69] to account for the natural interdependence that exists between parent and offspring data [70]. For all analyses, we first examined RP and then RRV_food_ and DD separately in order to explore how these relationships emerged behaviorally. Satterthwaite approximation was used to compute the test statistic, which allows for fractional degrees of freedom. All models were calculated using restricted maximum likelihood estimation and a first-order autoregressive covariance structure.

We visually examined each dependent variable histogram for skew on the control visit. RRV_food_ and, thereby, RP had a positive skew and were log transformed after adding 1 to all values in order to include non-responders (i.e., zero values). We also checked the linearity of each association by the visual examination of scatterplots. The relationship between RRV_food_ and age appeared to be nonlinear, with children having the greatest RRV_food_, adolescents having the smallest, and parents being in the middle. Thus, we used age group categories for the children and adolescents in later analyses. Covariates for each model were chosen based on the previous literature. For examinations of DD, these included age group [53], sex [71], and hunger [72]. For RRV_food_, the covariates were hunger [58], food liking [58], alternative activity liking, and the reinforcing value of the alternative activity (RRV_alt_) [73]. Models predicting RP included all of the above covariates. In the case of a significant interaction, we examined the simple slopes to understand which groups were significant within the levels of the other independent variables.

To test the hypothesis that adults with food insecurity would be more sensitive to acute financial losses and respond with greater RP, the appointment (level 1) was nested within the individual (level 2), using parent data only. The data were structured in a person-period dataset. This model included covariates, manipulated game winnings, offspring age group, food insecurity status, and the interaction between the manipulated game winnings, food insecurity status, and offspring age groups, as well as all possible two-way combinations of these. The same model was created for RRV_food_ and DD, using appropriate covariates for each.

To compare parents to their offspring in terms of RP across appointments, we included all participant data as well as a variable indicating the participant’s role in the dyad (parent vs. offspring). For this hypothesis, individuals’ data across appointments (level 1) were nested within families (level 2). The data were structured in a person-period pairwise dataset. To test the hypothesis that adolescents would be more similar, in terms of RP, to their parents than children would, the models included the covariates noted above, manipulated game winnings, offspring age group, dyad role, and the interaction between the offspring age group and dyad role. Again, the same model was created for RRV_food_ and DD, using appropriate covariates for each.

Finally, to examine the within-subject responses to the acute scarcity manipulations within family dyads and between those with and without food insecurity, we created the same dyadic models as above and included food security status and game winnings. We tested a four-way interaction of dyad role, offspring age group, food insecurity status, and game winnings along with all possible two-way and three-way combinations.

## 3. Results

### 3.1. Sample Characterization

Three hundred and twenty-seven parents completed the screening survey (CONSORT diagram available in Figure S1). Of these, 246 were determined to be eligible for participation in the study. In total, 9 of these families declined participation, 23 never responded to contact, and 106 were removed from eligibility because their recruitment subgroup was already full when they completed the screening survey. The remaining 108 families consented to participate in the study. The data from two families were excluded because it was revealed part-way through the appointments that they did not meet inclusion criteria. In one case, the child was a sibling of another participant (the data from the first enrolled child were retained), and in the other case, the parent revealed that the adolescent was taking stimulant medications. Of the remaining 106 families, 2 did not complete all three appointments. Because food insecurity was measured on the final appointment, we cannot be certain of which group they fell into. However, based on the screening survey, one family was receiving food assistance and one family was not. The available data for both of these families are included in all analyses. The RRV_food_ data from one appointment for two participants and from two appointments for one participant were excluded because the wrong food was administered during the appointment. Both the RRV_food_ and DD data were excluded from one appointment for four participants because the wrong (i.e., a duplicate) condition was administered. The DD data from one appointment for sixteen participants and from two appointments for four participants were excluded due to nonsystematic responses [64]. The remaining valid data for the participants from these and other appointments were included in all analyses. Participant characteristics can be found in Table 1. The final sample consisted of 53 families with an adolescent and 53 families with a child. Based on random assignment, 34% of the sample (N = 72) experienced the control visit first, followed by gain and loss; 35.4% (N = 75) were in the gain, loss, control order; the final 30.7% (N = 65) were in the loss, control, gain order of presentation. The parents of adolescents tended to be older, but there were otherwise no significant differences between the adolescent and child families.

As expected, the rate of food insecurity in our sample (20.8%) was greater than the national average (13.6%) [1]. Food insecurity status and food assistance participation were significantly related, such that 28% of food-secure families reported receiving food assistance in the last year, whereas 64% of food-insecure participants reported receiving this assistance (*Χ^2^* (2), N = 104) = 9.59, *p* = 0.002). Additionally, food-insecure households were more likely to have offspring who also reported food insecurity (*Χ*^2^ (2), N = 104) = 4.57, *p* = 0.033). The rate of food insecurity reported by the offspring in the sample was smaller than that for parents, at 13.4% (N = 14). The rates of food insecurity among the offspring were similar between adolescents (N = 6) and children (N = 8). In food-secure households, 10% of the offspring reported food insecurity compared with 27% of those in food-insecure households. There were no significant differences between food-secure families in terms of parental age, parental education, offspring sex, parent/offspring race (White vs. non-White), or parent/offspring ethnicity (all *p* > 0.05). Most of the parents/guardians who participated in the study were female (N = 100). The few male parents/guardians who participated in the study were more likely to report food insecurity (*Χ*^2^ (2, N = 104) = 7.23, *p* = 0.007). Food-secure and food-insecure families significantly differed in terms of household income (F(1, 90) = 18.79, *p* < 0.001) such that food-insecure parents reported lower income. Food-insecure parents also had significantly higher body mass indexes (F(1, 102) = 5.59, *p* = 0.020). Likewise, the offspring from food-insecure households had significantly greater body mass index z-scores (F(1, 102) = 8.24, *p* = 0.005).

### 3.2. Correlations

Zero-order relationships of the dependent variables and covariates between parents and offspring on the control appointment are presented in Table 2 (parents above the diagonal, offspring below the diagonal, and correlations within dyads on the diagonal). Among the dependent variables, the parent and offspring scores were positively related for RRV_food_ (*r*(100) = 0.33, *p* = 0.001). DD (reverse-scored) on the control visit was positively related to income (*r*(88) = 0.22, *p* = 0.035) and education (*r*(99) = 0.27, *p* = 0.006) among the parents.

### 3.3. Hypothesis Testing

#### 3.3.1. Food Insecurity, Acute Financial Scarcity, and Reinforcer Pathology in Adults

We tested the hypothesis that food-insecure parents would respond to financial losses with greater RP compared with financial gains. The first model, examining overall RP, showed a significant main effect of game winnings (*β* = −0.01, *t*(151.80) = −2.21, *p* = 0.029), which was moderated by food insecurity status (*β* = −0.03, *t*(151.97) = −2.70, *p* = 0.008). The simple slopes for this interaction revealed that food-insecure adults responded to financial losses by increasing RP (*β* = −0.03, *t*(154.15) = −2.69, *p* = 0.008), whereas food-secure adults showed no difference in RP based on financial gains or losses (*p* > 0.05).

When we broke RP into its two components and examined the same predictors, the model examining RRV_food_ revealed the same significant two-way interaction between food insecurity status and manipulated game winnings (*β* = −0.03, *t*(145.32) = −3.00, *p* = 0.003). The simple slopes for these interactions again showed that food=insecure adults increased their responding for food following financial losses (*β* = −0.02, *t*(137.68) = −2.39, *p* = 0.018). There was also a significant interaction between the offspring age group and game winnings (*β* = 0.41, *t*(92.35) = 0.77, *p* = 0.003), with simple slopes showing that parents of children increased their responding to food following financial losses. There were main effects of game winnings (*β* = −0.01, *t*(145.46) = −2.29, *p* = 0.024) and food liking (*β* = 0.02, *t*(240.90) = 3.39, *p* < 0.001).

For DD, which was reverse-scored, the results showed a significant effect of the visit number (*β* = 0.05, *t*(186.28) = 4.39, *p* = 0.000), which likely indicates practice effects across the appointments. Participants reporting greater hunger (*β* = −1.76 × 10^−3^, *t*(244.73) = −3.02, *p* = 0.003) had greater DD compared with those reporting less hunger. Finally, parents reporting food insecurity had greater DD compared with food-secure parents (*β* = −0.16, *t*(102.79) = −2.23, *p* = 0.028). However, there were no significant main effects or interactions with the game winnings for DD (all *p* > 0.05). Overall, these results suggest that financial losses increase the RRV_food_ among food-insecure parents, who already have greater DD, which results in an overall increase in RP.

#### 3.3.2. Associations between Parents and Offspring for RP

We hypothesized that, regardless of scarcity, adolescents would be more similar to their parents than children would in terms of RP. The model examining overall RP showed a significant interaction between dyad role and offspring age (*β* = 1.63, *t*(282.29) = 5.24, *p* < 0.001). There were also main effects of role (*β* = −1.22, *t*(393.02) = −6.35, *p* = 0.000), offspring age (*β* = −0.52, *t*(294.70) = −3.10, *p* = 0.002), and food liking (*β* = 0.02, *t*(502.98) = 3.48, *p* = 0.000). When we examined the simple slopes, we observed that adolescents did not differ from their parents in terms of RP (*p* > 0.05). Children, however, displayed significantly greater RP compared with their parents (*β* = −2.04, *t*(371.11) = −7.88, *p* = 0.000) and the adolescents (*β* = −1.34, *t*(501.26) = −5.75, *p* = 0.000).

When we broke RP into its two components, the model examining RRV_food_ also revealed a significant interaction between role and age group (*β* = 1.01, *t*(280.25) = 4.44, *p* < 0.001) as well as main effects for food liking (*β* = 0.01, *t*(526.70) = 4.62, *p* = 0.000), role (*β* = −0.24, *t*(316.63) = −2.11, *p* = 0.036), and offspring age group (*β* = −0.33, *t*(297.85) = −2.84, *p* = 0.005). Unexpectedly, the simple slopes for the interaction revealed a very similar pattern across the age groups to that of RP. The children exhibited greater RRV_food_ than the adolescents (*β* = −0.76, *t*(523.11) = −4.82, *p* = 0.000) and their own parents (*β* = −0.66, *t*(316.43) = −4.23, *p* = 0.000).

For DD, there were, again, significant main effects of the visit number (*β* = 0.03, *t*(279.89) = 2.21, *p* = 0.028) and dyad role (*β* = 0.25, *t*(414.78) = 7.47, *p* = 0.000). There was also a significant age group by dyad role interaction (*β* = −0.17, *t*(306.83) = −3.34, *p* = 0.001). In this case, the results followed typical developmental trajectories. Children had greater DD compared with adolescents (*β* = 0.09, *t*(526.48) = 2.46, *p* = 0.014) and their own parents (*β* = 0.32, *t*(386.53) = 7.29, *p* = 0.000). Adolescents also had greater DD compared with their parents (*β* = 0.17, *t*(339.29) = 4.10, *p* = 0.000). Overall, these data suggest that adolescents are more similar to their parents in terms of RP than children are, who have greater RP due to both greater DD and, unexpectedly, greater RRV_food_.

#### 3.3.3. Associations between Parents and Offspring Food Insecurity and Reactions to Acute Scarcity

**Figure 1:**
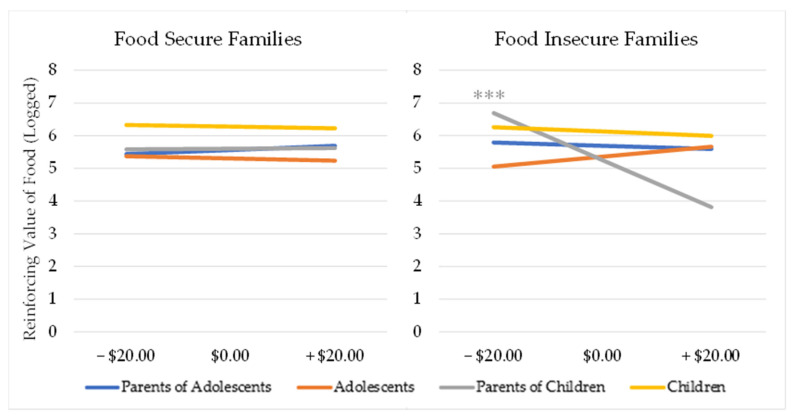
Changes in the RRV_food_ following Acute Scarcity. Note: Food insecure parents of children reacted to financial losses by increasing their RRV_food_. Scores for RRV_food_ are logged. *** = *p* < 0.001.

The results for the final hypotheses across families can be found in Table 3. We hypothesized that adolescents would be more similar to their parents than children in response to acute scarcity between food-secure and food-insecure households. For overall RP, there was a significant three-way interaction of dyad role, food insecurity status, and game winnings (*β* = −0.06, *t*(470.45) = −2.22, *p* = 0.027). The simple slopes for this interaction showed that financial losses preceded an increase in RP for food-insecure parents (*β* = −0.04, *t*(484.80) = −2.69, *p* = 0.007), regardless of offspring age group. At the same time, financial losses also preceded an increase in RRV_food_ among food-insecure families with children only, regardless of dyad role (*β* = −0.03, *t*(446.39) = −3.11, *p* = 0.002). Despite this, the four-way interaction of dyad role, offspring age group, food insecurity status, and game winnings was not significant (*p* > 0.05). Given this pattern of results, we also ran the simple slopes for the four-way interaction and found that, as expected, parents of children were the only group who reacted to the manipulation (*β* = −0.03, *t*(446.39) = −3.12, *p* = 0.002), (Figure 1). Overall, these data suggest that, after losing money, food-insecure parents experience an increase in their RRV_food_, an effect which is primarily driven by parents of children rather than parents of adolescents.

Food-insecure participants (i.e., across both age groups and dyads) had significantly greater DD compared with food-secure participants (*β* = −0.09, *t* (297.35) = −3.14, *p* = 0.002). The results from the above developmental model remained the same when we added in food insecurity and game winnings. There were no significant relationships with food insecurity or acute scarcity, suggesting that all offspring, regardless of age, were not more or less like their parents based on food insecurity in terms of DD. 

### 3.4. Post Hoc: Association between Parental Responses to Scarcity and Offspring RRV_food_

The above pattern of results led us to question why parents of children were particularly sensitive to the manipulation in terms of RRV_food_. We hypothesized that, because parents may wish to share the extra food with their offspring, the greater RRV_food_ among the children may have motivated the food-insecure parents of these children to earn additional food. To test this, we examined parents only and created a model that controlled for covariates and offspring age and tested the interaction between food insecurity status, manipulated game winnings, and the RRV_food_ for the offspring during that visit (i.e., matched on visit rather than condition) as well as all possible two-way interactions of these variables. Despite the significant correlation between the parent and offspring RRV_food_ noted in Table 2, the offspring RRV_food_ was not related to the parent RRV_food_ after controlling for covariates and the offspring age group. The offspring age group also did not interact with food insecurity and/or game winnings to predict the parents’ RRV_food_. This suggests that the differences in parent RRV_food_ in this study are not due to their child’s RRV_food_.

## 4. Discussion

This study showed that there is a consistent association between food insecurity and RP in adults, with complex relationships emerging within families. We replicated previous findings that adults with food insecurity have greater DD compared with food-secure adults [25,50]. Likewise, food-insecure parents experienced an increase in RP following financial losses. this change was primarily driven by an increase in their RRV_food_ after financial losses, which replicates previous findings [22,24]. In this study, however, this was primarily driven by parents of children, compared with parents of adolescents. Contrary to our hypothesis, this effect was not present for DD, which was unaffected by financial gains and losses in any model. Our hypothesis that parents and adolescents would be more similar to one another compared with parents and children in terms of RP was confirmed; however, this difference was due to both the expected decrease in DD as participants got older and the unexpected finding that children had greater RRV_food_ compared with adolescents and parents.

For food-insecure parents, the same small amount of money lost from our manipulation significantly increased their RRV_food_, particularly if they had a child in the study as opposed to an adolescent. By contrast, food-secure adults did not respond any differently for food regardless of financial gains or losses. This result replicates our previous findings among food-insecure adults [24] and suggests that the age of the child is an important driving factor of this relationship. In the design of this study, participants were able to take the food home with them at the end of the appointment. We hypothesized that parents might be gathering additional snacks to share with their children, who had greater RRV_food_ compared with adolescents and adults. However, we examined child RRV_food_ as a predictor of parent behavior and found no significant associations, which suggests that parents of children with a greater RRV_food_ were no more likely to earn additional food than those with a child with a lesser RRV_food_. We therefore suspect that financial losses do not just raise the RRV_food_ of the food-insecure parents in isolation but also raise their food demand for their children. Adolescents are likely to have more independence when it comes to gathering food [52] compared with children, and their parents may not be as sensitive to financial losses without a young child to feed every day. We also suspect that we did not see an effect of financial losses on the RRV_food_ among the adolescents and children because most of them were not directly experiencing food insecurity in the way that their parents were.

The relationships observed within family units and between age groups, regardless of food insecurity or acute financial losses, extend our understanding of RP among children and adolescents. Children displayed the greatest RP among all the groups, while adolescents and parents were lower on this scale and more similar to one another. DD is well understood to decrease through childhood and adolescence as the prefrontal cortex develops [74,75], which is reflected in our data. However, the strength of the relationship between age and RP was also driven by greater RRV_food_ among the children compared with the adolescents and parents. Individual RRV_food_ develops very early in the lifespan and is related to obesity risk among infants [76,77,78], children [61], and adolescents [79]. However, research has yet to examine how the RRV_food_ may change across developmental periods. These data are cross-sectional, and we cannot conclude that greater food reinforcement is a characteristic of middle childhood compared with adolescence. However, future research should examine the RRV_food_ over time to understand how it may change during development and to discover if childhood is a sensitive period for the development of this important obesity risk factor.

The overall financial situation of the home was associated with DD in this study. Our previous work in adolescents showed that parental education was positively related to adolescent DD [25]. Regardless of this, our manipulation of financial gains and losses had no effect on DD in any group in this study, which was contrary to our hypotheses and the previous literature, which showed an increase in present-thinking following the narrative priming of financial shock in adults [34]. Our manipulation used very small amounts of money (i.e., USD 5–20), and the DD task used larger amounts of hypothetical money (i.e., USD 50–1000). It is possible that the manipulation was too small to affect the preferences for larger amounts of money in the task or that real financial gains do not have a large effect on hypothetical financial gains. In a broader sense, it is also likely that the financial divisions in this income-stratified sample had a larger and more chronic effect on DD that is not easily altered by small monetary gains and losses.

The results presented here must be considered in the context of the participant sample that provided these data and the naturalistic and experimental nature of the study. Food insecurity was not experimentally assigned in this study, which limits the causal conclusions that can be drawn. Conversely, the engagement of families with food insecurity allows our results to be generalizable to the population of interest. This sample was also diverse in terms of race, ethnicity, and SES, but our participants were required to speak English. Therefore, these results may only be generalized to English-speaking families with food insecurity in the United States. Our staff worked to provide scheduling flexibility and free bus/train transportation to our participants. However, the number of appointments, the length of those appointments, and some procedures, such as fasting, likely amounted to a large burden for our participants, particularly those with limited financial resources. This burden likely affected the nature of our sample and may have amounted to a sample that is less disadvantaged than is typical for food-insecure families in the United States. In this study, we allowed the participants to take the food home with them to remain consistent with our previous study in this area. However, doing so likely changed the behavior of the participants, and some of the relationships we are seeing may be due to a motivation to store or share the study foods rather than a motivation to eat them immediately. Relatedly, we included one child from each family but did not account for the ages of siblings, which, based on our results among the parents, could have affected the parents responding for food. Future research should investigate these differences in behavior to better determine how scarcity affects motivation for one’s own food intake versus those of others in the family unit.

The current study has many strengths. The data presented here are experimental, and we used real money for the manipulation, which is less common for questions related to financial scarcity. This dataset is also dyadic, which allows for a new layer of investigation as to how parents and their offspring operate within a family unit. We also recruited a sample that was diverse in terms of SES, allowing us to make a broad set of comparisons both between and within participants. This sample is also balanced in terms of race and ethnicity, which adds assurance that the variables in question are not confounded with race/ethnicity. Finally, the engagement of families with food insecurity allowed us to see how this experience is associated with eating behavior directly. However, the results of this study must be considered in the context of its limitations. In recruiting for this study, efforts were made to conceal the research questions as well as to ensure participant comfort during screening, which made it more difficult to recruit food-insecure families. We chose to measure food insecurity after the participants were already enrolled and, instead, recruit based on the receipt of food assistance. This approach appropriately protected participants’ privacy and comfort as well as the research questions but also failed to recruit as many food-insecure families as planned. A study that directly recruits food-insecure families may be better powered to explore these questions more deeply and examine more moderators than we were able to do. Relatedly, because half of this sample was either below or near the poverty line, there is an increased risk that our participant payment could have been coercive to this vulnerable population. To address this risk, the compensation amount was chosen to go no further than an appropriate reimbursement for the participants’ time and energy, and payment for each appointment was tied to attendance rather than the completion of study activities. However, we have no way of determining if the compensation for this study may have altered the behavior of our vulnerable families. Despite these limitations, we believe that the strong experimental design of this study and careful attention to participant comfort has allowed us to learn more about the effects of financial losses on reinforcer pathology among families with food insecurity.

## 5. Conclusions

When taken together, the current results suggest that RP is deeply entwined with financial scarcity, with complex interactions across family units and across development. Food insecurity was related to greater DD across all age groups and all manipulations. RP was very high among children, regardless of economic background, and the parents of these children were greatly affected by food insecurity, making them more sensitive to small financial gains and losses in terms of their RRV_food_. Although parents and adolescents were more similar in terms of RP, particularly in the case of the RRV_food_, their responses to both food insecurity and acute financial losses were not entwined with one another. These findings suggest that the effects of food insecurity on childhood eating may be strongest in middle childhood and may be primarily driven by parent behavior. Future inquiry into this topic will benefit from longitudinal investigation to better understand how RP changes over the course of development as well as how financial scarcity may change those developmental trajectories. At the policy level, this study further evidences the harms of food insecurity and suggests that programs to alleviate food insecurity must provide sufficient resources for both the parents and children in the household to mitigate its harmful effects.

## Figures and Tables

**Table 1:** Sample Characteristics (N = 106 families).

Variable	Adolescents (N = 53)	Children (N = 53)	*p*	Variable	Adolescents (N = 53)	Children (N = 53)	*p*
Offspring Sex, *n* (%)			0.56	Household Safety Net Benefits, *n* (%)			
Female	27 (50.90)	30 (56.60)		Any Benefits	17 (32.10)	20 (37.70)	0.24
Male	26 (49.10)	23 (43.40)		WIC	6 (11.30)	10 (18.90)	0.30
Offspring Race/Ethnicity, *n* (%)			0.62	School Lunch Program	1 (1.90)	2 (3.80)	0.56
Black/African American	11 (20.80)	14 (26.40)		Food Bank Donations	5 (9.40)	7 (13.20)	0.54
White	31 (58.50)	32 (60.40)		SNAP	10 (18.90)	12 (22.60)	0.63
Other/Multiracial	11 (20.80)	7 (13.20)		Offspring zBMI, M (SD)	0.94 (0.92)	0.66 (1.08)	0.16
Hispanic or Latinx	10 (18.90)	4 (7.50)	0.09	Parent Age, mean (SD)	46.15 (11.21)	37.63 (9.54)	0.00
Household Income (USD), *n* (%)			0.28	Parent BMI, mean (SD)	25.67 (6.13)	19.23 (5.00)	0.35
Less than 25,000	7 (13.30)	8 (15.20)		Parent DVs/Covariates on Control Visit, M (SD)			
25,000 to 49,999	10 (18.80)	7 (13.20)		RP	1348.83 (5047.49)	962.17 (2241.62)	0.62
50,000 to 74,999	14 (26.40)	6 (11.30)		RRV_food_	235.69 (312.08)	263.46 (425.39)	0.71
75,000 to 99,999	7 (13.20)	6 (11.30)		RRV_Alt_	161.57 (270.08)	148.08 (150.15)	0.75
100,000 or greater	11 (20.80)	16 (30.20)		DD	0.51 (0.31)	0.57 (0.28)	0.30
Parent Education, *n* (%)			0.16	Hunger	36.25 (26.91)	39.96 (27.65)	0.52
≤High School Diploma	15 (28.30)	6 (11.30)		Food Liking	57.14 (23.57)	64.82 (20.11)	0.08
Certificate	3 (5.70)	1 (1.90)		Alternative Liking	62.86 (18.11)	63.94 (19.87)	0.78
Associate Degree	12 (22.60)	11 (20.80)		Offspring DVs/Covariates on Control Visit, M (SD)			
Bachelor’s Degree	12 (22.60)	20 (37.70)		RP	6018.78 (23,252.72)	7318.18 (11,659.63)	0.73
Postgraduate Degree	10 (18.90)	12 (22.60)		RRV_food_	387.31 (640.97)	459.23 (424.91)	0.50
Household Food Insecurity, *n* (%)			0.22	RRV_Alt_	180.00 (402.25)	264.23 (284.62)	0.22
High Food Security	35 (66.00)	35 (66.00)		DD	0.38 (0.32)	0.28 (0.32)	0.15
Marginal Food Security	8 (15.10)	4 (7.50)		Hunger	51.23 (26.38)	60.38 (32.64)	0.13
Low Food Security	9 (17.00)	10 (18.90)		Food Liking	65.29 (20.92)	81.81 (20.73)	0.00
Very Low Food Security	0 (0.00)	3 (5.70)		Alternative Liking	62.06 (20.40)	74.02 (30.37)	0.02

Note: Special Supplemental Nutrition Program for Women, Infants, and Children (WIC), Supplemental Nutrition Assistance Program (SNAP), dependent variables (DVs).

**Table 2:** Correlations on Control Visit.

	DD	RRV_food_	RP	S	Age	Hung	FL	AL	RRV_alt_	Win
Delay Discounting (DD)	(−0.09)	−0.13	−0.55 ***	−0.10	−0.08	−0.13	−0.01	−0.25 *	−0.19	0.01
RRV of Food (RRV_food_)	0.02	(0.33 **)	0.88 ***	−0.10	−0.07	0.31 **	0.29 **	0.07	0.12	−0.17
Reinforcer Pathology (RP)	−0.57 ***	0.73 ***	(0.19)	−0.03	−0.04	0.33 **	0.26 *	0.15	0.17	−0.17
Sex (S)	−0.05	−0.16	−0.10	(0.08)	−0.15	0.13	−0.10	−0.03	0.04	0.08
Age (Age)	0.15	−0.23 *	−0.25 *	0.07	(0.38 ***)	−0.17	−0.34 ***	−0.25 *	−0.01	−0.11
Hunger (Hung)	0.12	0.28 **	0.06	0.00	−0.21 *	(0.23 *)	0.49 ***	0.18	0.25 *	−0.03
Food Liking (FL)	−0.02	0.25 *	0.17	0.14	−0.37 ***	0.46 ***	(0.14)	0.54 ***	0.10	0.04
Alternative Liking (AL)	0.00	0.21 *	0.12	−0.04	−0.24 *	0.17	0.31 **	(0.05)	0.40 **	0.13
RRV of Alternative (RRV_alt_)	−0.09	0.37 ***	0.29 **	−0.05	−0.31 **	0.32 **	0.14	0.38 **	(0.11)	0.11
Game Winnings (Win)	−0.07	0.12	0.12	0.01	−0.14	−0.12	−0.06	0.24 *	0.09	(0.14) *

Note: Parents above the diagonal, offspring below. In parentheses are parents and offspring’s correlations with one another. *** = *p* <.001, ** = *p* < 0.01, * = *p* < 0.05.

**Table 3:** Effects of Financial Losses on Reinforcer Pathology across Families and Food Insecurity Status.

	Reinforcer Pathology	Reinforcing Value of Food	Delay Discounting (AUC)
	β (SE)	β (SE)	β (SE)
Intercept	6.58 (0.16) ***	5.10 (0.10) ***	0.38 (0.02) ***
Visit	−0.16 (0.10)	−0.06 (0.07)	0.04 (0.02) *
Sex	0.09 (0.21)	--	−0.04 (0.04)
Hunger	3.03 × 10^−3^ (0.00)	3.40 × 10^−3^ (0.00)	−6.4 × 10^−4^ (0.00)
Food Liking	0.01 (0.00) **	0.01 (0.00)***	--
Alternative Liking	3.69 × 10^−3^ (0.00)	8.10 × 10^−4^ (0.00)	--
RRV Alternative	0.05 (0.04)	0.05 (0.03)	--
Role (Mom vs. Offspring)	−1.22 (0.23) ***	−0.21 (0.14)	0.24 (0.04) ***
Offspring Age	−0.73 (0.22) **	−0.27 (0.15)	0.03 (0.03)
Role X Age	2.10 (0.40) ***	0.93 (0.27) **	−0.21 (0.07) **
Game Winnings (Win)	−0.01 (0.01)	−0.01 (0.00)	−1.39 × 10^−5^ (0.00)
Role X Win	−0.02 (0.01)	−0.02 (0.01) *	8.57 × 10^−5^ (0.00)
Age X Win	0.03 (0.01) **	0.02 (0.01) *	−2.66 × 10^−4^ (0.00)
Role X Age X Win	0.01 (0.03)	0.02 (0.02)	2.16 × 10^−3^ (0.00)
Household Food Insecurity	0.21 (0.22)	−0.08 (0.15)	−0.09 (0.03) **
Food Insecurity X Role	0.08 (0.39)	0.06 (0.27)	−0.02 (0.07)
Food Insecurity X Age	−0.54 (0.43)	0.32 (0.29)	0.09 (0.07)
Food Insecurity X Win	−0.02 (0.01)	−0.01 (0.01)	−7.17 × 10^−5^ (0.00)
Food Insecurity X Role X Age	1.37 (0.79)	0.32 (0.54)	−0.12 (0.14)
Food Insecurity X Role X Win	−0.06 (0.03) *	−0.05 (0.02) **	6.17 × 10^−4^ (0.00)
Food Insecurity X Age X Win	0.04 (0.03)	0.04 (0.02) *	−2.78 × 10^−3^ (0.00)
Food Insecurity X Role X Age X Win	0.03 (0.05)	0.02 (0.04)	−4.41 × 10^−3^ (0.01)

Note: *** = *p* <.001, ** = *p* < 0.01, * = *p* < 0.05.

## Data Availability

The data presented in this study are available on request from the corresponding author. The data are not publicly available due to privacy concerns.

## Supplementary Materials

The following supporting information can be downloaded at: https://www.mdpi.com/article/10.3390/children9091338/s1, Figure S1: CONSORT diagram.

## Abbreviations

RRV	Relative Reinforcing value
DD	Delay Discounting
RP	Reinforcer Pathology
SES	Socioeconomic Status
WIC	Special Supplemental Nutrition Program for Women, Infants, and Children
SNAP	Supplemental Nutrition Assistance Program
