# Supplementary figures and images for: The Effect of Financial Scarcity on Reinforcer Pathology: A Dyadic Developmental Examination
Source: Children (Basel). 2022 Sep 1;9(9):1338. doi: 10.3390/children9091338 (PMC9498192; doi:10.3390/children9091338)

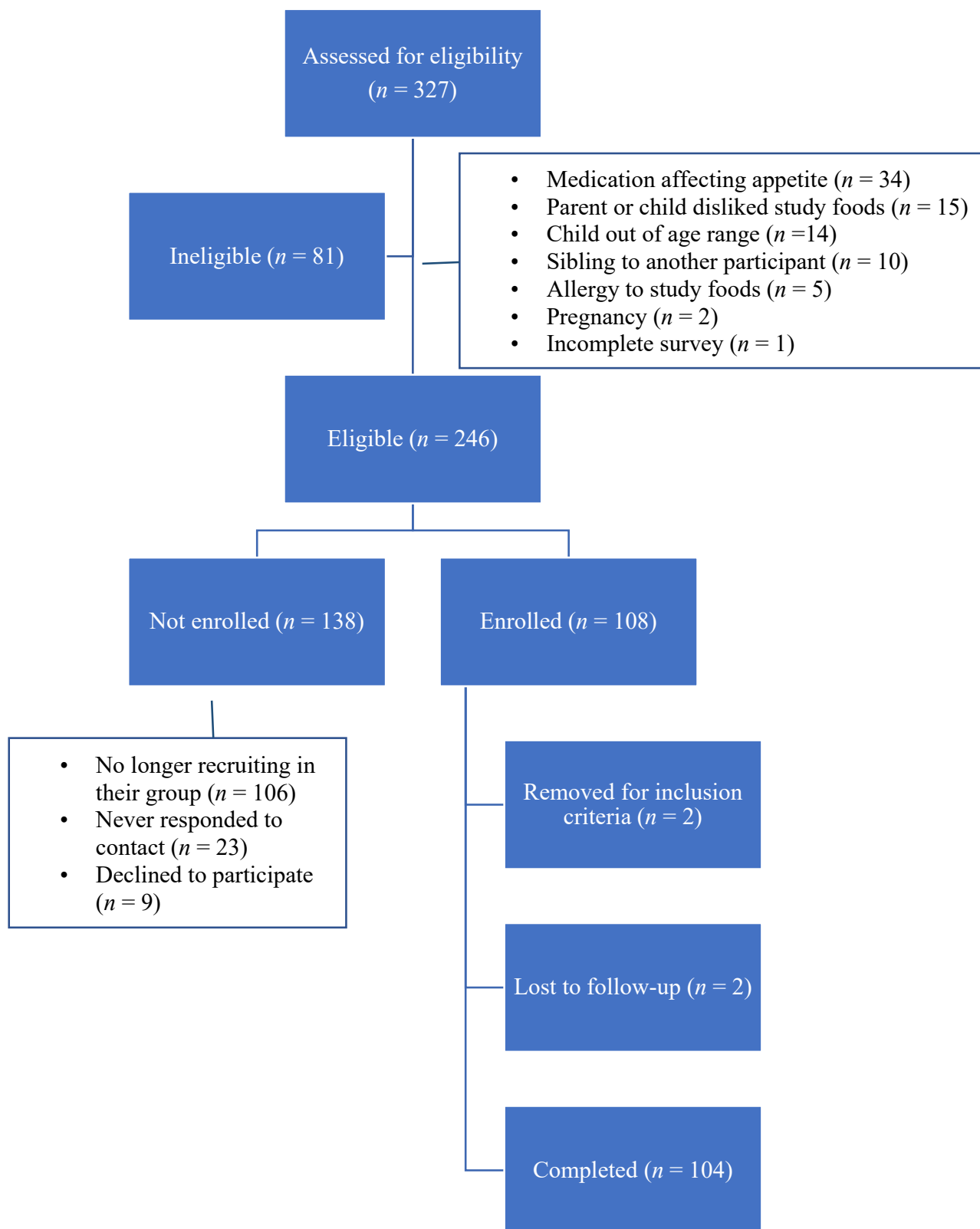

**Figure S1.** CONSORT diagram.

Supplement: Supplementary file 1 [file children-09-01338-s001.zip › children-1878717-supplementary.pdf]
